# Effects of Wi-Fi (2.45 GHz) Exposure on Apoptosis, Sperm Parameters and Testicular Histomorphometry in Rats: A Time Course Study

**DOI:** 10.22074/cellj.2016.3740

**Published:** 2015-07-11

**Authors:** Saeed Shokri, Aiob Soltani, Mahsa Kazemi, Dariush Sardari, Farshid Babapoor Mofrad

**Affiliations:** 1 Department of Anatomical Sciences, Faculty of Medicine, Zanjan University of Medical Sciences (ZUMS), Zanjan, Iran; 2 Department of Nuclear Engineering, Science and Research Branch, Islamic Azad University, Tehran, Iran; 3 Department of Physiology, Faculty of Medicine, Zanjan University of Medical Sciences, Zanjan, Iran

**Keywords:** Apoptosis, Electromagnetic Radiation, Testis, Spermatogenesis

## Abstract

**Objective:**

In today’s world, 2.45-GHz radio-frequency radiation (RFR) from industrial, scientific, medical, military and domestic applications is the main part of indoor-outdoor electromagnetic field exposure. Long-term effects of 2.45-GHz Wi-Fi radiation on male reproductive system was not known completely. Therefore, this study aimed to investigate the major cause of male infertility during short- and long-term exposure of Wi-Fi radiation.

**Materials and Methods:**

This is an animal experimental study, which was conducted in the Department of Anatomical Sciences, Faculty of Medicine, Zanjan University of Medical Sciences, Zanjan, IRAN, from June to August 2014. Three-month-old male Wistar rats (n=27) were exposed to the 2.45 GHz radiation in a chamber with two Wi-Fi antennas on opposite walls. Animals were divided into the three following groups: I. control group (n=9) including healthy animals without any exposure to the antenna, II. 1-hour group (n=9) exposed to the 2.45 GHz Wi-Fi radiation for 1 hour per day during two months and III.7-hour group (n=9) exposed to the 2.45 GHz Wi-Fi radiation for 7 hours per day during 2 months. Sperm parameters, caspase-3 concentrations, histomorphometric changes of testis in addition to the apoptotic indexes were evaluated in the exposed and control animals.

**Results:**

Both 1-hour and 7-hour groups showed a decrease in sperm parameters in a time dependent pattern. In parallel, the number of apoptosis-positive cells and caspase-3 activity increased in the seminiferous tubules of exposed rats. The seminal vesicle weight reduced significantly in both1-hour or 7-hour groups in comparison to the control group.

**Conclusion:**

Regarding to the progressive privilege of 2.45 GHz wireless networks in our environment, we concluded that there should be a major concern regarding the timedependent exposure of whole-body to the higher frequencies of Wi-Fi networks existing in the vicinity of our living places.

## Introduction

Electromagnetic radiation (EMR) from different sources, such as microwave ovens, radar, satellite links, wireless communication, frequency modulation (FM) radio and television (TV) transmitters/ antennas, is the main part of indoor- outdoor electromagnetic field exposure spectrum (1, 2). Widespread usage of industrial, scientific, medical, military and domestic applications with 2.45-GHz radio-frequency radiation is inevitable in today’s world. As the Wi-Fi technology is low cost and operates in the unlicensed spectrum at 2.40-2.4 GHz, the leakage of Wi-Fi radiation into the environment is unavoidable (3, 4).

It has been suggested that male infertility during the past several decades is related to the direct or indirect exposure to certain environmental factors such as radio-frequency electromagnetic waves (RF-EMW) (5, 6). The effects of 2.45-GHz EMR on reproductive system have already been shown (7-10). Kumar et al. (11) showed 2.45 GHz microwave exposure causes an increase in caspase-3 and creatine kinase activities in the sperm in addition to a decrease in plasma levels of testosterone and melatonin in the exposed rat. *In vitro* study by Avendano et al. (12), focused on the effect of Wi-Fi radiation on the motility reduction and DNA fragmentation of human spermatozoa. The negative effect of Wi-Fi emitting RF-EMW has been also reported on the *ex vivo* human sperm parameters (13), sexual behavior (14) and testis structure of exposed animals (15). It is believed that exposure to EMR can enhance production of reactive oxygen species (ROS) (9, 12, 15-18). An increase in lipid peroxidation levels in addition to a decrease in antioxidant enzymes and vitamin A and E levels (11, 19) can explain some aspects of 2.45-GHz EMR effect on reproductive tissues of male rats. Kim et al. (20) showed that the effect of exposure to 2.45-GHz EMR on proliferation and differentiation of spermatogonia is correlated with serum sex hormone level. In parallel with defect in spermatogenesis process, the negative effects of 2.45-GHz EMR on histopathological changes and apoptosis status of rat testis are inevitable (7). Nowadays 2.45 GHz wireless networks have become much more commonplace in our environment (21). Wireless devices have been widespread used in our living and working environments for longer exposure times than wireless phones which may have an untoward influence on health (2). According to the Bioinitiative Report (http://www.bioinitiative.org/), current safety guidelines for electromagnetic field (EMF) exposure are not sufficient and should be revised based on data from various toxicological tests (22). Due to whole body exposure to the RF-EMR, we tried to analyze potential effects of 2.45 GHz Wi-Fi radiation from a wireless antenna on the reproductive system of freely moving male rats for short- and long-term. Indeed, the consequences of exposure to the emitted radiofrequency waves from Wi-Fi antenna were the major concerns of the present study.

## Materials and Methods

### Animals

This is an animal experimental study, which was conducted in the Department of Anatomical Sciences, Faculty of Medicine, Zanjan University of Medical Sciences, Zanjan, Iran, from June to August 2014. Animals, 3-month old Wistar strain rats (n=27), were maintained as national guidelines and protocols approved by the Institutional Animal Ethics Committee (IAEC no.03/028/07).

All experimental protocols were approved by the Ethics Committee of Zanjan University of Medical Sciences, Zanjan, Iran. Healthy adult male albino rats weighing 250 g, were randomly selected and housed under environmentally controlled conditions. The rats were fed with a standard laboratory diet (Pars Dam Co., Tehran, Iran) and clean drinking water *ad libitum*.

### Exposure system

The exposure system was a chamber (180 cm×80 cm×70 cm), designed for whole-body exposure of free-moving rats to a Wi-Fi signal. Two Wi-Fi antennas (NanoStation Loco M2, 2.45 GHz, 8.5 dBi, Ubiquiti Networks, Inc. USA) were placed at the center of two sides of the chamber. A previous study applied a restrainer to fix space between antenna and rat (19). Since it was a stressful condition that could probably affect hormonal balance of animals, we tried to assess the effect of radiation on the free moving animals (14, 23).

Animals were divided into three following groups (n=9 per each group): I. control group including healthy animals without any exposure to the antenna, II. 1-hour group including animals exposed to the 2.45 GHz Wi-Fi radiation one hour per day during two months (1 hour/day/2 months) (7, 14, 20) and III. 7-hour group including animals exposed to the 2.45 GHz Wi-Fi radiation seven hours per day during two months (7 hours/day/2 months). All exposure conditions were coded and analyzed in a blind manner.

### Laboratory studies, body and reproductive organ weights

Animals were anesthetized intraperitoneally with a mixture of ketamine (45 mg⁄kg, Sigma- Aldrich, Germany) and xylazine (35 mg⁄kg, Sigma Aldrich, Germany). The weight gain of animal in each group was defined as the differences between initial and final body weights. The reproductive organs including testes, epididymis, seminal vesicles and ventral prostate were accurately weighed following being dissected out from surrounded adipose and connective tissues by an expert anatomist. The relative weights of each dissected reproductive organ were expressed as the weight of organ to the body weight ratio. The samples of testicular tissues were fixed in a 4% buffered formaldehyde solution (Merck, Germany) and then were embedded in paraffin wax (Merck, Germany) using standard techniques for preparing 5-μm thick sections. Other side testicle was randomly dissected out and transferred to a cryotube for storing in liquid nitrogen in order to determine the caspase-3 activity.

### Sperm characteristics

Caudal part of epididymis was dissected out and chopped in the 5 ml of Ham’s F10 medium solution (GIBCO, USA). Epididymal sperm were collected following 5 minutes incubation at 37˚C to allow sperm to swim out of the epididymal tubules. One drop of sperm suspension was placed on a microscope slide and cover slipped. At least 10 microscopic fields were observed at ×40 magnification by a phase contrast microscope (Olympus BX51, Tokyo, Japan). The sperm motility parameters were recorded according to the World Health Organization (WHO) recommendations. The percentages of progressive, motile, and immotile sperm were expressed as the ratio to the total counted sperm. The sperm count parameters were also obtained by the method described in the WHO recommendations (24). Briefly, 5 μl aliquot of epididymal sperm was diluted with 95 μl of diluents (0.35% formalin containing 5% NaHCO_3_ and 0.25% trypan blue, Merck, Germany), and approximately 10 μl of this diluted specimen was transferred to the counting chambers of the haemocytometer. The cells were counted with a light microscope at ×40 magnification. For morphological abnormalities, sperm smears were drawn on slides and allowed to air-dry overnight. Slides were stained with 1% eosin- Y⁄5% nigrosin (Merck, Germany) and examined at ×40 magnification. Amorphous, hook less, bicephalic, coiled or abnormal tails were considered as the morphological abnormalities (25). The total percentages of abnormal and normal sperm were then calculated.

### Histopathological evaluation of spermatogenesis

Either the number of germinal cell layers or Johnson’s score were measured for categorizing spermatogenesis in the testes. According to Miller et al. (26) description, the number of germinal epithelial layers was counted in 10 seminiferous tubules. Based on Johnson’s method, a score of 1-10 was applied for each cross-sectioned tubule (27).

### Apoptosis in reproductive tissues of rats

Germ cell apoptosis was evaluated by terminal deoxynucleotidyl transferase (TdT) enzymemediated dUTP nick end labeling (TUNEL) assay kit (Roche, Germany). Briefly, 5-μm thick paraffinembedded sections were microwave-pretreated in 10 mM citrate buffer (Merck, Germany, pH=6.0) for 10 minutes. Sections were incubated with blocking solution (3% H_2_O_2_ in methanol, Merck, Germany) for 10 minutes, then were washed with phosphate-buffered saline (PBS, Merck, Germany). The specimens were incubated with TUNEL reaction mixture (TdT and nucleotide mixtures in reaction buffer) at 37˚C for 60 minutes. Finally the slides were stained with converter-POD (antifluorescein antibody, Fab fragment from sheep, conjugated with horse-radish peroxidase-POD) for 30 minutes.

The 3, 3΄- Diaminobenzidine (DAB) substrate (Roche, Germany) was applied for color development. TUNEL positive cells exhibited a brown nuclear stain. In each group, the number of stained cells was counted in 100 seminiferous tubules. The number of stained germ cells was counted. Apoptotic index-1 (AI-1) was defined as the number of apoptotic TUNEL-positive cells per 100 tubules and apoptotic index-2 (AI-2) as the number of tubules containing apoptotic cells per 100 tubules. All of measurements were performed by an expert technician who was blinded to the experiment procedure.

### Caspase-3 activity assay

Briefly, lysis buffer at pH=7.5, including 10 mM Tris-HCL, 10 mM NaH_2_PO_4_/NaHPO_4_, 130 mM NaCl, 1% Triton-X100 and 10 mM NaPPi, all materials were purchased from Merck products-Germany that were added to the testes tissue samples and lysates were incubated at 4˚C for 20 minutes. The lysates were centrifuged at 14000 rpm and stored in liquid nitrogen for further analysis. Next 100 ml proteins from lysates were incubated with Ac-DEVD-pNA in a 96-well plate at 37˚C for 1 hour, and colorimetric substrate (DEVD-AFC, Biomol, Plymouth Meeting, PA, USA) was preferentially cleaved by caspase- 3. The amounts of 7-amino-4-methyl-coumarin (AMC) were monitored 1 hour with a plate reader (Anthos2020, USA) and absorption was measured, normalized to the absorbance of time zero and expressed as percent of control.

The data were expressed as mean ± standard errors of the mean (SEM). The variables were analyzed by one-way ANOVA. When a significance found, Tukey post hoc tests were performed. All analyses were performed using the SPSS (SPSS Inc., Chicago, IL, USA) version 16. The statistical significance level was set at P≤0.05.

## Results

**Fig.1:**
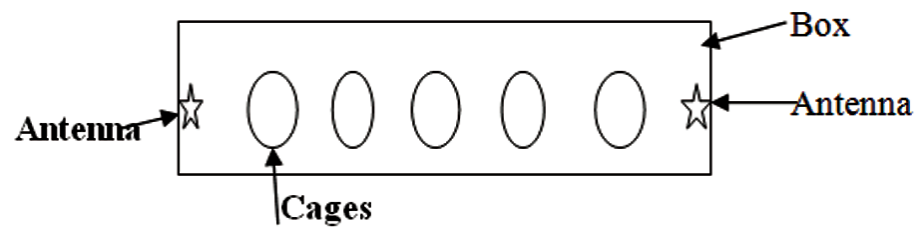
Represents a schematic picture of designed apparatus as the exposure system. Box dimension was 180 cm×80 cm×70 cm. Two Wi-Fi antennas (NanoStation Loco M2, 2.45 GHz, 8.5 dBi, Ubiquiti Networks, Inc. USA) were placed at the center of two sides of the chamber. Animal cages were placed between two antennas.

Table 1 shows two months exposure of animals to the 2.45 GHz Wi-Fi radiation in the designed exposure apparatus (Fig.1), indicating no significant changes in the body weight of both 1- and 7-hour groups.

Despite right and left seminal vesicles, 1 hour and 7 hours chronic exposure caused no significant changes in the relative weight of testicles or other accessory sex organs. The relative weight of both right and left seminal vesicles reduced significantly (P≤0.001) following two months chronic exposure of animals to the 2.45 GHz Wi-Fi radiation either for 1 hour per day or 7 hours per day (Table 1).

**Table 1:** The effect of chronic exposure to the 2.45 GHz Wi-Fi radiation on the weights of testis, epididymis, prostate and seminal vesicle in mature male rats


Groups	Relative righttestisweight (%)	Relative lefttestisweight (%)	Relative rightepididymisweight (%)	Relative leftepididymisweight (%)	Relative rightSeminal vesicleweight (%)	Relative leftSeminal vesicleweight (%)	RelativeVentral prostateweight (%)	Weightgain (g)

Control	0.43 ± 0.01	0.45 ± 0.01	0.19 ± 0.0	0.18 ± 0.0	0.18± 0.01	0.16± 0.01	0.18± 0.01	59.37 ± 3.83
1-hourgroup	0.42 ± 0.01	0.43 ± 0.01	0.18 ± 0.0	0.18 ± 0.0	0.08± 0.0^1^	0.08± 0.01^1^	0.19± 0.0	40.11 ± 8.90
7-hourgroup	0.41 ± 0.01	0.42 ± 0.01	0.17 ± 0.0	0.17 ± 0.0	0.08± 0.02^1^	0.09± 0.0^1^	0.22± 0.01	50.54 ± 6.88


Values are expressed as mean ± s tandard errors of the mean (SEM). Chronic exposure to the 2.45 GHz Wi-Fi r adiation caused significant differences in the relative weights of both right and left seminal vesicles. The right and left seminal vesicles column were compared with the control group. 1-hour group; exposed to the 2.45 GHz Wi-Fi radiation one hour per day during two months (1 hour/day/2 months). 7-hour group; exposed to the 2.45 GHz Wi-Fi radiation seven hour per day during two months (7 hour/day/2 months).^1^; P value≤0.001.

**Fig.2:**
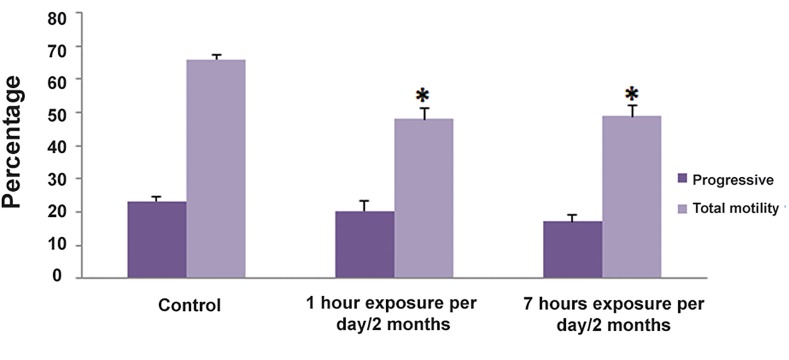
The effect of chronic exposure to the 2.45 GHz Wi-Fi radiation on total percentage of progressively motile sperm. Values are expressed as mean ± standard errors of the mean (SEM). *; P value≤ 0.001.

We examined the proportion of the different sperm motility grades as shown in figure 2. Two months exposure to the 2.45 GHz Wi-Fi radiation caused significant changes on the sperm motility parameters (Fig.2). Although the percentage of progressive sperm showed no significant differences in the experimental groups, the percentages of total motility parameters, considered as the percentage of progressive and motile sperm, reduced significantly in both 1- and 7-hour groups. Therefore, our findings showed a significant reduction in the percentage of motile sperm in 1-hour (27.75 ± 1.27 vs. 44.89 ± 0.81, P≤0.001) and 7-hour (31.87 ± 1.58 vs. 44.89 ± 0.81, P≤0.001) groups as compared to control group.

Table 2 shows that chronic exposure to the 2.45 GHz Wi-Fi radiations showed a clear negative impact on the concentration parameters. Sperm samples from both 1-hour (P≤0.001) and 7-hour groups (P≤0.05) exhibited a significant lower concentration as compared to the control group. In parallel with the sperm count reduction, the proportion of normal to abnormal sperm showed a similar reduction in the both 1- and 7-hour groups.

**Table 2:** The effect of chronic exposure to the 2.45 GHz Wi-Fi radiations on the concentration parameters


	Control	1-hour group	7-hour group

Sperm count (×10^6^/ml)	3.49 ± 1.72	2.16 ± 2.77^2^	2.68 ± 1.261
Normal sperm (%)	87.34 ± 1.05	78.66 ± 1.22^2^	83.68 ± 0.901


Values are expressed as mean ± standard errors of the mean (SEM). The 1-hour and 7-hour groups were compared to the control ones. 1-hour group: exposed to the 2.45 GHz Wi-Fi radiation one hour per day during two months (1 hour/day/2 months). 7-hour group: ex-posed to the 2.45 GHz Wi-Fi radiation seven hour per day during two months (7 hour/day/2 months). ^1^; P≤0.05 and ^2^; P≤0.001.

**Fig.3:**
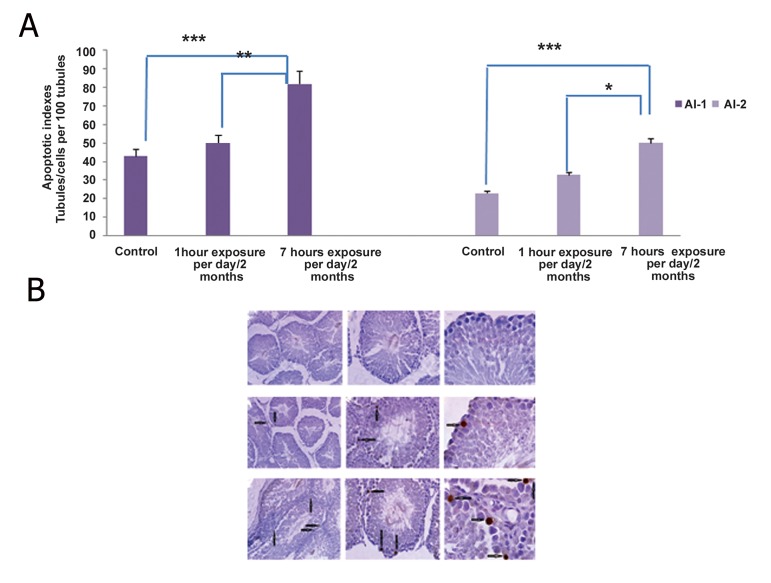
A. The effect of chronic exposure to the 2.45 GHz Wi-Fi radiation on the apoptotic indexes as either number of apoptotic terminal deoxynucleotidyl transferase (TdT) enzyme mediated dUTP nick end labeling (TUNEL)-positive cells per 100 tubules (AI-1) or the number of tubules containing apoptotic cells per 100 tubules (AI-2). Values are expressed as mean ± standard errors of the mean (SEM). The 1-hour and 7-hour groups were compared to the control ones. ***; P≤0.001, **; P≤0.01 and *; P≤0.05 and B. The effect of chronic exposure to the 2.45 GHz Wi-Fi radiation on the apoptosis of spermatogenic cell line. Control group (a. magnification ×10, b. magnification ×40 and c. magnification ×100). 1-hour group (d. magnification ×10, e. magnification ×40 and f. magnification ×100). 7-hour group (g. magnification ×10, h. magnification ×40 and i. magnification ×100). (scale bar=100 μm) arrows show apoptotic cells.

**Fig.4:**
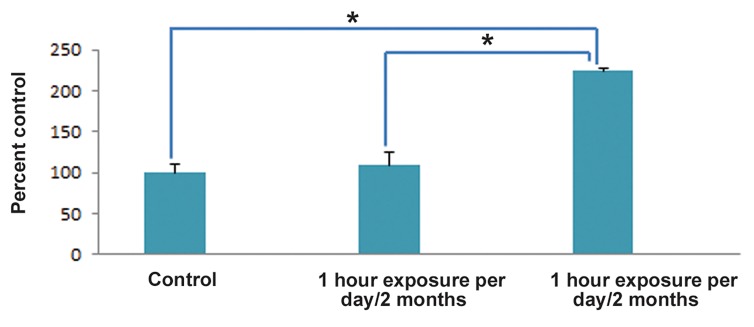
The effect of chronic exposure to the 2.45 GHz Wi-Fi radiation on the concentration of caspase-3. Values are expressed as mean ± standard errors of the mean (SEM). *; P≤0.001.

Table 3 shows that the 1-hour group exposed to the 2.45 GHz Wi-Fi radiations demonstrated a normal architecture of the seminiferous tubules and interstitial tissue. The germinal epithelium of testis was intact with an average thickness of about five cell layers. On the contrary, 7-hour group exposed to the 2.45 GHz Wi-Fi radiations caused a significant decrease in both the number of germ cell layers (P≤0.01) and the mean testicular score (P≤0.001). Quantitative and descriptive analysis of TUNEL stained slides in figure 3A and B respectively, show that in parallel with the significant reduction in both the number of germ cell layers and the Johnson’s criteria of the 7-hour group, evaluation of apoptotic indexes showed a significant increase in the either the number of apoptotic cells (P≤0.001) or positive tubules per 100 tubules (P≤0.001) in the same group. As it is shown in the figure 4, the increased level of caspase-3 can be a good explanation for testicular apoptosis occurring in the testis of 7-hour animals. Interestingly, lack of significant differences in the number of germ cell layers and the mean testicular score of 1-hour group was accompanied with lack of significant criteria in apoptotic indexes and the caspase-3 concentration. However, two experimental groups showed a significant differences in apoptotic indexes, caspase 3 activity and Johnson’s criteria.

**Table 3:** The Effects of chronic exposure to the 2.45 GHz Wi-Fi radiations on apoptosis of spermatogenesis


	Johnson’scriteria	Number ofgerm cell layers

Control	9.48± 0.14	5.58 ± 0.08
1-hour group	9.24± 0.05	5.61± 0.05
7-hour group	8.75± 0.06^2^*	5.25± 0.05^1^*


Values are expressed as mean ± standard errors of the mean (SEM). The 1-hour and 7-hour groups were compared to the control ones. ^1^; P≤0.01, ^2^; P≤0.001 and ^*^; The comparison be-tween 1-hour and 7-hour groups. 1-hour group: exposed to the 2.45 GHz Wi-Fi radiation one hour per day during two months (1 hour/day/2 months). 7-hour group: exposed to the 2.45 GHz Wi-Fi radiation seven hour per day during two months (7 hour/ day/2 months). P≤0.001.

## Discussion

Decline in male fertility, as one of parameters in this study, is considered as a major concern during the past several decades. It has been suggested that direct or indirect exposure to RF-EMW as the main environmental factor plays a dominant role in the observed decline (28). The 2400-2500 GHz radio frequency emitting from Wi-Fi-enabled devices has a long exposure time over a very wide area (2, 19, 21). Hence, this transmitted energy can be absorbed by human body (8, 29).

No deleterious effects of 2.4 GHz Wi-Fi exposure on the body weight and reproductive organ weights were observed in the either 1- or 7-hour groups; however, exposure effect on the seminal vesicle weights was observed. This present result is in line with previous reported animal experiment that demonstrated no adverse effects of 2.45 GHz radio-frequency exposure on the body weight (14) as well as testis and prostate weights (15, 19). Interestingly, 1 hour and 7 hours exposure caused a decline in seminal vesicles weight in comparison to related value of the control group. Although there is no previous report indicating the deleterious effect of 2.45 GHz radiation on seminal vesicles, khaki et al. (30) showed that 50 Hz non-ionizing radiation during two months caused a decrease in seminal vesicles weight. It is noted that epithelial cell proliferation in the seminal vesicles is testosterone-dependent (31). It has been shown that RF-EMF exposure probably reduces the serum testosterone in experimental animals (32, 33).

Alternatively, deficiency in blood testosterone can alter epithelial proliferation in the seminal vesicles. Specifically, Kumar et al. (11) showed that long-term exposure of 2.45 GHz radiation from microwave source can reduce the level of serum testosterone in rats. Consequently, we speculated that the reduced seminal vesicle weight following 2.45 GHz exposure is likely to be related to the reduction of serum testosterone in rats.

Some evidences have indicated that sperm abnormalities are frequent following exposure to RF-EMW (34, 35). We found that sperm concentration, motility and morphology were affected significantly by exposure to the 2.45 GHz RFR from a Wi-Fi antenna. The observed effects were dependent on the longevity of exposure per day. Recent *in vitro* pilot studies on the effect of exposure of the 2.45 GHz RFR on human ejaculated semen found changes in the motility and DNA fragmentation of exposed sperm (12, 13). Kim et al. (20) found no significant reduction in the epididymal sperm count after exposure of rats to the 2.45 GHz EMF [a designed magnetron (Samsungelectronics, Korea) operating at 2.45 GHz by Institute of Biomedical Engineering, Yeungnam University, Daegu, Korea] for 1 hour or 2 hours per day during 8 weeks. Moreover, they reported no abnormal morphology in the exposed groups.

It was also shown that microwave radiation decreases the sperm count (20). A plausible explanation for the impaired sperm motility could be induced oxidative stress by RF-EMW from Wi-Fi devices (12). Oxidation of phospholipids, as a major component in the sperm mitochondrial sheath (36), can disturb mitochondrial membrane potential which causes high levels of ROS to be released into the cytoplasm, leading to deplete the energy supply and to affect both sperm motility and kinetics (37, 38). Peroxidation of unesterified polyunsaturated fatty acids in the cell membrane of spermatozoa can lead to cell death as well (39). However, an in vitro pilot study by Oni et al. (13) showed that 1 hour exposure of 2.45 RFR from a laptop antenna (a 2.4 GHz picostation by Ubiquity Networks, USA) had no effects on sperm concentration and sperm head, whereas tail and middle piece defect were evitable following exposure to the RFR. The negative effect of chronic RF exposure from cell phones on the count and the quality of sperm was also reported in the previous researches (40, 41). Interestingly, the negative correlation between both abnormal structure and decreased motility of sperm with the longevity of exposure to the RFR from mobile phones was showed by Wdowiak et al. (42). It is believed that EMF, especially extremely low frequency, induces free radical production that is responsible for sperm deformities (43). Although, the mechanism of cascade is unknown, it has been recently demonstrated that depletion in the activity of both histone kinase and protein kinase may serve as a measure of microwave EMF’s ability to affect spermatogenesis and cell cycle in sperm (8).

In the testis tissue of the animals exposed to 7 hours of 2.45 GHz Wi-Fi radiation for 60 days, the number of germinal cell layers (5.25 ± 0.05 vs. 5.58 ± 0.08, P≤0.01) and Johnson’s score (8.75 ± 0.06 vs. 9.48 ± 0.14, P≤0.001) showed a significant reduction as compared to control group. In parallel, the profound DNA damage in 7-hour group was accompanied with an increase in the activity of caspase-3. In accordance with these findings, several authors focused mainly on the destructive effects of RFR on the germinal cell layers of male reproductive organ (11, 14, 15, 19-20, 32, 34, 41). It is shown that 2.45 GHz microwave radiation decreases the diameters of seminiferous tubule (41, 44). Saygin et al. (7) showed changes in histopathology and apoptosis status of rat testis under exposure to 2.45-GHz EMF, at 3.21 W/kg specific absorption rate for 60 minutes/day for 28 days.

On the other hand, Poulletier de Gannes et al. (14) found no microscopic lesions in the testes of male Wistar rats by exposing animals to the 2450 MHz Wi-Fi signal (1 hour/day, 6 days/week, 0.08 and 4 specific absorption rate). Moreover, Kim et al. (20) showed that both the measured diameter of seminiferous tubule and average Johnson’s score of testicular biopsy did not change significantly by exposure to the 2.45 GHz EMF (1 hour or 2 hours per day/8 weeks, 1.41W/Kg and 60.1 mV/m electric field intensity. Although they observed no significant difference in the number of spermatids, a significant difference was seen in the number of spermatocytes between the control and exposed group. Atasoy et al. (15) applied standard wireless gateways (2.437 GHz, 24 hours a day for 20 weeks) and their results showed that median values of testicular biopsy score, using Johnson’s scale, were significantly lower in the exposed than the control group. They attributed the occurrence of DNA damage to the decreased levels of catalase and glutathione peroxidase activity as a consequence of 2.45 GHz RF that led to induce oxidative stress. Apoptosis is induced by ROS through cytochrome C and caspases-3 and -9 which in turn leads to a high rate of single and double DNA strand break (45). Actually, caspase-3 is a key mediator of apoptosis (46).

It is showed that 2.45-GHz microwave exposure (2 hours per day/ 2 months) increases caspase and creatine kinase activities and decreases melatonin level in the testes of exposed rats (11). The role of 2.45-GHz EMF in inducing oxidative stress by enhancing the lipid peroxidation, free radical formation and modifying antioxidant systems has been approved previously (19, 47, 48). Interestingly, the 2.45 GHz induced oxidative stress was attributed to the reduced levels of testosterone and non-enzymatic antioxidants such as vitamin A and E (19, 32).

## Conclusion

High frequency, specifically 2.45 GHz Wi-Fi radiation, induces a decrease in sperm parameters along with an increase in apoptosis-positive cells and caspase-3 activity in the seminiferous tubules of Wistar rats, specially in 7-hour group. It reduced seminal vesicle weight following 2.45 GHz exposure. Considering the progressive privilege of 2.45 GHz wireless networks in our environment, we concluded that there should be a major concern about the time-dependent exposure of our body to the higher frequencies of Wi-Fi antenna.
